# A Treatise on the Sympathetic Relation between the Stomach and the Brain; and Throughout, between the Digestive and the Nervous Systems, in the Causation and Cure of Diseases

**Published:** 1841-04-01

**Authors:** 


A TREATISE ON THE SYMPATHETIC RELATION BETWEEN THE STOMACH AND THE BRAIN; AND, THROUGHOUT, BETWEEN THE DIGESTIVE AND THE NERVOUS SYSTEMS, IN THE CAUSATION AND CURE OF DISEASES. With an Appendix, containing a few Observations on certain Points connected with the Treatment of Chronic Disease, and its attendant Debility. By *Charles Wightman*, M.D. Licentiate of the Royal College of Physicians of London, and Resident Physician in Newcastle-upon-Tyne. London, Simpkin, Marshall & Co. 1840. Small Octavo, pp. 192.

IT has been said " Medicina nusquam non est,"—an aphorism expressive at once of the universality and antiquity of our art. No less universal, and scarcely less ancient, is the doctrine of sympathy betwixt the stomach and the brain. It is, in fact, one of the most familiar, as well as most fundamental doctrines of physic ; and has been taught from time immemorial ; although, perhaps, it has been looked upon as too much of a truism to be specially treated of in monographic essays. We do not say this, in any degree, from a desire to be over-critical with Dr. Wightman, or to disparage his favourite subject of contemplation. Far otherwise! We freely bear witness to the talent, as well as diligence, he has displayed in illustrating this sympathetic relation, which he justly considers has such an important pathological and practical bearing. It is to the originality of his researches that we demur,—not to their value or usefulness. There is a little prudery, we think, in so well-informed a writer ransacking old bibliographical catalogues, and citing the almost forgotten works of Veegens, Rega, and Rahn, as the only ones bearing upon his subject. In this, as in some other instances, we suppose—

" 'Tis distance lends enchantment to the view ;"

for on no other hypothesis can we account for our author's thus reversing Time's telescope, and overlooking what has been written, and well written, on the doctrine of sympathy, by later and even contemporary authors. It

---

is not for us to speak of the writings of the Editor of this Journal, except in modest and restrained terms. But delicacy need not altogether prevent us from referring those whom it may concern to the various works of Dr. James Johnson, for copious and forcible illustrations of this very doctrine, viz. the intimate sympathy subsisting betwixt the brain and stomach, on the one hand; and betwixt the digestive functions and the influence of the nerves, on the other. We might quote his Treatise on "Civic Life, Sedentary Habits, and Intellectual Refinement," published more than twenty years ago. We might also quote his striking chapter on the "Morbi Eruditorum, or Diseases of Literature," at p. 143 of his book on "The Influence of the Atmosphere," published in the year 1818. Or, finally, we might express our surprise that the graphic picture of the *patho-proteian malady*, (as Dr. Johnson calls it, in his "Economy of Health," published so lately as 1837,) should have escaped the notice of a gentleman of Dr. Wightman's literary proficiency, and studious habits. But, waiving this strain of discussion, as one in which we may be supposed to have too near a personal concern, we may inform Dr. W. that his favourite subject of "Sympathy" is pretty fully treated of in Bichât's "Anatomie Generale;" and that Ploucquet's "Literatura Medica Digesta," under the heads "Consensus" and "Sympathia," supplies a whole host of preceding authorities.* It would be foreign to our purpose to go more at length into this matter, as we are contending for truth rather than for victory.

It is time, however, to enter more particularly into the contents of Dr. Wightman's book, and to examine them more in detail. After a well-written Preface and Introduction, he proceeds to discuss the influence of the stomach upon the brain; and of the brain upon the stomach. He illustrates this "sympathetic relation"—1. By the consequences of Injuries to the Head. 2. By the Effects of external Violence on the Epigastrium. 3. By Fever, more especially Typhus. 4. Acute Gastritis. 5. Apoplexy. 6. Epilepsy. 7. Hydrocephalus. 8. Chronic Disease of the Brain. 9. Sick-headache. 10. Dyspepsia and Nervous Disorder combined. 11.

---

* Even non-professional writers are no strangers to this sympathetic doctrine. Shakespeare, who by the wonderful power of his intuitive genius, seems to have known everything, was well aware of the influence of the depressing passions (acting, of course, through the brain and nerves,) in suspending the function of the stomach. Cardinal Wolsey, on first discovering his loss of Court favour, is made to bewail his altered fortunes, in the following beautiful and touching terms:—

"———— nay then, farewell!
I have touch'd the highest point of all my greatness;
And, from that full meridian of my glory,
I haste now to my setting;—I shall fall
Like a bright exhalation in the evening,
And no man see me more!"

The King, in announcing to the Cardinal the loss of his royal countenance, and the disgrace sure to ensue, does it in the following words: a physiologist or pathologist could not have expressed it better:—

"———— And now to breakfast, with
What appetite you may!"

Mental Exercise. 12. The Operation of certain Poisons. To the above is added a section in vindication of the late Dr. Hamilton's plan of treating various acute and chronic diseases by purgative medicines; and an Appendix on the influence of minute doses of mercury, in the treatment of chronic disease, according to the instructions of Dr. Wilson Philip. This simple enumeration will convey some idea of the *practical* interest and importance of the work.

As familiar examples of the influence of sympathy between the brain and stomach, Dr. W. gives the following:—

"When a person previously in the full enjoyment of health, and possessed of great strength of body, receives a severe blow upon the head,—in addition to the abolition of sense and motion, (the direct effect of the concussion of the brain,) vomiting in general almost immediately takes place; and not only are the contents of the stomach itself rejected, but a quantity of bile, of a dark green colour, entirely different from the healthy appearance of this fluid, is thrown up. Most individuals who have been unaccustomed to sailing, while affected, on going to sea, with certain indications of disordered brain, such as vertigo, anxiety, and prostration of strength, are also seized with nausea and vomiting, the symptoms of disordered stomach; more especially if the weather be tempestuous, and consequently much tossing of the vessel upon the waves. There are some persons, also, whose nervous system is so irritable that they experience the same symptoms of stomach disorder from dancing for any length of time; from riding in a carriage, particularly if they be drawn backwards; from swinging; and from turning their bodies in rapid gyration. In all these cases, it is evident that the morbid affection of the stomach is only secondary,—or the consequence of this organ sympathizing with the brain; the functions of which are in the first place disordered by the blow inflicted upon the head, the motion of the carriage, the tossing of the vessel upon the waves, and the rapid circuitous movement of the body; indeed, in the latter instances, this is sufficiently demonstrated by the vertiginous sensation primarily induced. This disorder of the functions of the brain occurs in these cases, although there is no lesion or disorganization of its structure; but solely from its commotion excited by the causes mentioned; by which commotion the nervous influence is disturbed—interrupted at its source; a suspension of the balance between the brain and the stomach consequently takes place, and the latter by sympathy manifests the disorder of its functions. On the other hand, a common example of morbid affection of the brain, arising from sympathy with the stomach as the organ primarily disordered, is presented to our notice by the effects of the indigestion of aliment. It is undeniable that if food, either difficult of digestion, or in too great quantity, be received into the stomach, not only will this organ be oppressed, as shewn by its own proper symptoms of nausea and vomiting; and a quantity of sordes will be accumulated in the bowels, occasioning diseases in these; but also headache, vertigo, diminution and depravation of sight in various ways, deafness, noises in the head, and confusion of the internal senses, the well-known indications of cerebral disorder, will be induced;—all which symptoms will disappear on the offending matters being discharged from the stomach by the operation of emetics; and from the bowels, by that of purgatives." Pp. 2—4.

Of this explanation, we shall only say that it is good, according to the present reigning doctrines; but that, in point of fact, it explains nothing:

"Felix qui potuit rerum cognoscere causas."

In tracing the arcana of nature, we are too apt to mistake words for things!

We speak of sympathy in physiology, just as we speak of attraction or repulsion in physics. The word, in either case, simply expresses a *law:* it does not explain the *power* whose mysterious movements constitute that law. The language of science does not advance us one jot towards the fountain-head of knowledge. This is mortifying to the pride of intellect. But yet it is the best and most salutary sort of knowledge to know that we are ignorant; or, in other words, to be aware how little we know of the mysterious agencies going on within and around us. In what we have just said as to the defectiveness of his explanation, we need scarcely say that we do not consider the defect chargeable to Dr. Wightman; but rather to the present imperfection of our knowledge; or, perhaps, of our faculties.

The space to which our remarks have already extended, makes it impossible for us to notice, even cursorily, the different chapters into which our author's excellent little work is sub-divided. We must therefore content ourselves with recommending them generally. We are quite sure that a careful perusal of the whole book will be useful to many, and acceptable to all; and we sincerely wish for it a rapid and extensive sale. But we cannot conclude without telling Dr. Wightman, great as is our respect for his practical talents and judgment, that we cannot go along with him in his out-and-out admiration of Dr. Hamilton's purgative practice. The eminent founder of that practice was himself, we *know*, a little too indiscriminate in its application; and, as was almost to have been expected, his admiring imitators have been still more so. The plan, from its apparent simplicity and energy, possessed great attractions for the young, the indolent, and the innovating; but we believe there are few—very few—whose opinions have been matured and chastened by experience, that still cling to the practice, or continue it to the same extent as inculcated by Dr. Hamilton.

As little can we acquiesce in the sanction which Dr. Wightman has given to the paramount utility of minute doses of mercury in the treatment of chronic disease; a practice enjoined by Dr. Wilson Philip. For that distinguished physician and physiologist we entertain very high respect:—we also think most favourably of Dr. Wightman. But nevertheless we retain our own opinion, founded on experience; and beg to differ from them both. "Amicus Socrates, amicus Plato; sed magis amica veritas." We have found, in our own practice, that the method of minute doses will not do. And our experience, in this instance, is confirmed by that of others; for we remember, a few years back, when Dr. Wilson Philip's book on the subject first came out, we put queries to the most enlightened of our medical brethren as to the result of their experience on this point. They were unanimous in condemning the practice of minute doses, as being at once inert, and unsatisfactory. Nor is this the sole objection. So much time is required to achieve success in those instances where success follows the "minute" practice, that the physician is ever exposed to the suspicion, either of the patient or of the bye-standers, that he is procrastinating the cure for mercenary purposes;—a suspicion than which none can be more galling to a man of feeling and of probity; and against which the "conscia mens recti" is no adequate counterpoise. We may also observe that those who find the "minute-dose" system to answer, must have ways and means of securing the confidence and stability of their patients to which we are strangers. We constantly find, that where a case hangs long in hand, the patient, or his friends become fidgetty and unsettled—if not dissatisfied. The upshot is, that long ere the "minute-dose" plan, or any other plan which requires *time* for its element, has had a fair chance of succeeding, the patient is off, at rail-road speed, to place himself under the infallible care of some fortunate juggler at some fashionable watering-place!

